# Supplementary material for: Knowledge, attitudes, and practices (KAPs) regarding tick-borne rickettsial disease among medical staff in endemic areas of China
Source: PeerJ. 2024 Jun 18;12:e17562. doi: 10.7717/peerj.17562 (PMC11192025; doi:10.7717/peerj.17562)
Supplement: Supplemental Information 1 [file peerj-12-17562-s001.docx]

**医务人员蜱传立克次体病知识态度行为**

**调查表**

尊敬的朋友，您好！

感谢您回答这份调查问卷。我们是市疾控中心工作人员，我们想了解一下您对蜱传立克次体病的知识以及一些相关的行为方式的认知。您的真实回答有助于我们认识和理解当前人们的认知情况和行为态度，为今后能更好的普及健康知识，针对不同群众制定合理的宣传方式和行为引导提供相关依据。调查所涉及到的个人及家庭资料部分，我们将依法予以保护。感谢您的参与和支持！

**一、一般信息**

1.您的姓名：

单位名称：

2.性别：

①男 ②女

4.您的年龄：

①30岁以下 ②30-39岁 ③40-49岁 ④50岁及以上

5.您的文化程度：

①中专及以下 ②大专 ③本科 ④研究生及以上

6.您所在的单位为：

①三级医院 ②二级医院 ③乡镇卫生院 ④村卫生室 ⑤疾控中心

7.您的科室：

①急诊 ②门诊 ③感染科 ④皮肤科 ⑤内科 ⑥外科 ⑦检验科 ⑧其他：_____

8.您的工作年限：

①10年以下 ②10-20年 ③21-30年 ④30年以上

9.您的职业

①临床医师 ②护士 ③检验人员 ④公共卫生医师 ⑤其他：_____

1. 您的职称

①无职称 ②初级 ③中级 ④副高 ⑤正高

11.您是否接受过关于蜱传立克次体病防控知识相关的培训

①是 ②否

1. **认知**

1.蜱传立克次体病病人主要症状和体征是什么？（可多选）

①发热 ②头痛 ③皮疹 ④乏力 ⑤焦痂 ⑥淋巴结肿大

2.蜱传立克次体病的常见潜伏期一般为多少天？

①1-3天 ②7-14天 ③21天

3.蜱传立克次体病是人畜共患类疾病吗？

①是 ②不是 ③部分是

4.下列哪种情况容易感染蜱传立克次体病？（可多选）

①足球场踢足球 ②户外采茶 ③耕种 ④打猎 ⑤游泳

5.蜱传立克次体病治疗一般需要持续多久？

①1-3天 ②5-7天 ③10天以上

6.按照美国疾病预防控制中心的建议，蜱传立克次体病的抗菌治疗首选哪项药物？

①强力霉素 ②氯霉素 ③大环内酯类 ④利福平

7.下列哪项属于蜱传立克次体病？（可多选）

①发热伴血小板减少综合征 ②斑疹伤寒 ③斑点热 ④新疆出血热 ⑤恙虫病

⑥埃立克次体病

8.蜱传立克次体病高发季节是？

①全年高发

②温暖的季节高发（4-9月）

③寒冷的季节高发（1-3月，9-12月）

9.人被蜱虫叮咬后，该如何处理？

①用干净的细尖头镊子将蜱虫尽可能靠近皮肤表面夹住，以稳定、均匀的力向上拉动

②用手直接将蜱虫取出

③用干净的细尖头镊子将蜱虫尽可能靠近皮肤表面夹住，迅速拉出蜱虫

④不作处理，待蜱虫吸饱血自动脱落

**三、态度**

1.我认为蜱传立克次体病是一个重要的公共卫生问题

①非常不同意 ②不同意 ③无法确定 ④同意 ⑤非常同意

2.我认为蜱传立克次体病需要采取积极的预防措施

①非常不同意 ②不同意 ③无法确定 ④同意 ⑤非常同意

3.我认为接诊过程中，应积极佩戴医用外科口罩和手套、穿防护服

①非常不同意 ②不同意 ③无法确定 ④同意 ⑤非常同意

4.在从事临床/护理工作中，我认为医生/护士需要接受立克次体病相关业务培训

①非常不同意 ②不同意 ③无法确定 ④同意 ⑤非常同意

5.我认为立克次体病经有效治疗能完全治愈

①非常不同意 ②不同意 ③无法确定 ④同意 ⑤非常同意

6.我会主动去了解立克次体病疫情和诊疗的最新动态

①非常不同意 ②不同意 ③无法确定 ④同意 ⑤非常同意

7.我很愿意担负起给患者和家属普及立克次体病个体防护相关知识的职责

①非常不同意 ②不同意 ③无法确定 ④同意 ⑤非常同意

**四、行为**

1.工作期间，我会严格按规定每4 小时更换一次外科口罩

①非常不同意 ②不同意 ③无法确定 ④同意 ⑤非常同意

2.工作期间，接触患者皮肤、血液或体液的操作时我都会佩戴手套、眼罩

①非常不同意 ②不同意 ③无法确定 ④同意 ⑤非常同意

3.当病人出现不明原因发热、皮疹时，我会考虑可能是立克次体病

①非常不同意 ②不同意 ③无法确定 ④同意 ⑤非常同意

4.当病人出现蜱传立克次体病可疑暴露史时，我会积极询问其流行病学史

①非常不同意 ②不同意 ③无法确定 ④同意 ⑤非常同意

5.当病人出现蜱传立克次体病可疑症状时，我会立即进行采样检测

①非常不同意 ②不同意 ③无法确定 ④同意 ⑤非常同意

6.当病人确诊为立克次体病时，我会积极向医院院感部门报告

①非常不同意 ②不同意 ③无法确定 ④同意 ⑤非常同意

7.若伤口不小心触碰到立克次体病病人的分泌物，我会立即用水冲洗，酒精消毒

①非常不同意 ②不同意 ③无法确定 ④同意 ⑤非常同意
