# Supplementary material for: Knowledge, attitudes, and practices (KAPs) regarding tick-borne rickettsial disease among medical staff in endemic areas of China
Source: PeerJ. 2024 Jun 18;12:e17562. doi: 10.7717/peerj.17562 (PMC11192025; doi:10.7717/peerj.17562)
Supplement: Supplemental Information 2 [file peerj-12-17562-s002.docx]

**Survey of knowledge, attitude and behavior of medical staff on tick-borne Rickettsial disease**

Dear friends, hello!

Thank you for completing the survey. We represent the City CDC, and we are interested in gaining insights into your understanding of tick-borne rickettsial disease and associated behavioral patterns. Your honest responses will assist us in comprehending the current cognitive landscape and behavioral dispositions among the populace. This information serves as a valuable foundation for enhancing future health education efforts and devising tailored publicity strategies and behavioral guidelines for diverse individuals. Please be assured that the personal and familial data collected during this investigation will be safeguarded in accordance with the law. Thank you for your valuable participation and unwavering support.

1. general information
2. Your name:
3. Unit name:
4. Gender: ① male ② female
5. Your age: ① under 30 years old ②30-39 years old ③40-49 years old ④50 years old and above
6. Your education level: ① secondary school and below ② junior college ③ undergraduate ④ postgraduate or above
7. Your unit is: ① tertiary hospital ② secondary hospital ③ township health center ④ village clinic ⑤ CDC
8. Your department: ① emergency treatment ② outpatient service ③ infectious department ④ dermatology ⑤ internal medicine ⑥ surgery ⑦ clinical laboratory ⑧ other: _____
9. Your years of service: ① less than 10 years ②10-20 years ③21-30 years ④ more than 30 years
10. Your occupation:①clinicians ② nurses ③ laboratory personnel ④ public health physicians ⑤ others: ____
11. Your title: ① No title ② Junior ③ Intermediate ④ Deputy senior ⑤ Senior

11. Have you received any training on tick-borne disease prevention and control?: ① Yes ② No

1. Cognition
2. What are the main signs and symptoms of tick-borne rickettsial disease?(Multiple choices are possible) ① fever ② headache ③ rash ④ fatigue ⑤ eschar ⑥ lymph node enlargement
3. How many days is the common incubation period of tick-borne rickettsial disease?①1-3 days ②7-14 days ③21 days
4. Is tick-borne rickettsial disease a zoonotic disease?① yes ② no ③ Part yes

4.Which of the following is susceptible to tick-borne rickettsial disease?(Multiple choices are possible) ①football field playing football ② outdoor tea picking ③ farming ④ hunting ⑤ swimming

5. How long does tick-borne rickettsial disease treatment usually last?①1-3 days ②5-7 days ③ more than 10 days

6. According to the recommendations of the Centers for Disease Control and Prevention, which drug is the first choice for the antibacterial treatment of tick-borne rickettsial disease? ① doxycycline ② chloramphenicol ③ macrolides ④ Rifampicin

7. Which of the following is tick-borne rickettsial disease?(Multiple choices are possible) ① fever with thrombocytopenia syndrome ② typhus fever ③ spot fever ④ xinjiang hemorrhagic fever ⑤ tsutsugamushi disease ⑥ ehrickettsial disease

8.What is the season for tick-borne rickettsial disease?① high incidence throughout the year ② high incidence in warm season (April to September) ③ high incidence in cold season (January to March, September to December)

9.What should I do if I am bitten by a tick?① hold the tick as close to the skin surface as possible with clean, fine-pointed tweezers and pull it upward with a steady, even force ② remove the tick directly with your hand ③ hold the tick as close to the skin surface as possible with clean, fine-pointed tweezers and quickly pull the tick out ④ without treatment, the tick will automatically fall off when it is full of blood

三、Attitude

1. I think tick-borne rickettsial disease is an important public health problem. ① strongly disagree ② disagree ③ uncertain ④ agree ⑤ strongly agree

2.I think tick-borne rickettsial disease needs to take active preventive measures. ① strongly disagree ② disagree ③ uncertain ④ agree ⑤ strongly agree

3.In my opinion, surgical masks, gloves and protective clothing should be worn during the treatment. ① strongly disagree ② disagree ③ uncertain ④ agree ⑤ strongly agree

4.In clinical/nursing work, I think doctors/nurses need to be trained in tick-borne rickettsial disease. ① strongly disagree ② disagree ③ uncertain ④ agree ⑤ strongly agree

5.I think tick-borne rickettsial disease can be completely cured with effective treatment. ① strongly disagree ② disagree ③ uncertain ④ agree ⑤ strongly agree

6.I will take the initiative to learn the latest developments of tick-borne rickettsial disease epidemic and diagnosis and treatment ① strongly disagree ② disagree ③ uncertain ④ agree ⑤ strongly agree

7.I am willing to take on the responsibility of educating patients and families about tick-borne rickettsial disease individual protection knowledge ① strongly disagree ② disagree ③ uncertain ④ agree ⑤ strongly agree

四、behavior

1.During the working period, I will strictly change the surgical mask every 4 hours according to the regulations. ① strongly disagree ② disagree ③ uncertain ④ agree ⑤ strongly agree

2.During the work period, I will wear gloves and eye masks when I come into contact with patients' skin, blood or body fluids. ① strongly disagree ② disagree ③ uncertain ④ agree ⑤ strongly agree

3.When the patient has an unexplained fever or rash, I will consider the possibility of tick-borne rickettsial disease ① strongly disagree ② disagree ③ uncertain ④ agree ⑤ strongly agree

4.When a patient has a suspected history of exposure to tick-borne rickettsial disease, I will actively inquire about their epidemiological history. ① strongly disagree ② disagree ③ uncertain ④ agree ⑤ strongly agree

5.When a patient shows suspicious symptoms of tick-borne rickettsial disease, I immediately take a sample for testing. ① strongly disagree ② disagree ③ uncertain ④ agree ⑤ strongly agree

6.When the patient is diagnosed with tick-borne rickettsial disease, I will actively report to the hospital sensory department.① strongly disagree ② disagree ③ cannot confirm ④ agree ⑤ strongly agree

7. If the wound accidentally touches the secretions of tick-borne rickettsial disease, I will immediately rinse with water and disinfect with alcohol. ① strongly disagree ② disagree ③ cannot confirm ④ agree ⑤ strongly agree
